# Cultural Value Orientations and Alcohol Consumption in 74 Countries: A Societal-Level Analysis

**DOI:** 10.3389/fpsyg.2017.01963

**Published:** 2017-11-20

**Authors:** Richard A. Inman, Sara M. G. da Silva, Rasha R. Bayoumi, Paul H. P. Hanel

**Affiliations:** ^1^Centro de Investigação em Psicologia para o Desenvolvimento, Universidade Lusíada – Norte Porto, Porto, Portugal; ^2^School of Psychology, Cardiff University, Cardiff, United Kingdom; ^3^Department of Psychology, University of Bath, Bath, United Kingdom

**Keywords:** alcohol, culture, values, risk factors, public health psychology

## Abstract

A significant proportion of all deaths globally can be attributed to alcohol consumption. Although a range of correlates of alcohol consumption have already been identified at the individual level, less is understood about correlates at the macro level, such as cultural values. As a development in this understanding may prove useful for global health organizations aiming to tackle the problems associated with excessive drinking, our aim was to investigate the association between encultured alcohol consumption and Cultural Value Orientations. We obtained data describing average alcohol consumption and Cultural Value Orientations, for 74 countries, from an online data repository. To assess whether Cultural Value Orientations are associated with alcohol consumption we calculated partial correlations and performed a ridge regression analysis. Our analyses revealed that Cultural Value Orientations were significantly associated with alcohol consumption, even after controlling for average income and education level. A profile emerged in which values of autonomy and harmony were shown to be positively associated with alcohol consumption, and hierarchy and embeddedness negatively associated with alcohol consumption. The effect was modified by gender. Changes in cultural Harmony, Mastery, Autonomy and Egalitarianism were associated with increases in alcohol consumption in males, but not females, while changes in cultural Embeddedness and Hierarchy were associated with decreases in consumption in females, but no change in males. Finally, we demonstrate that latitude, and by extension its covariates such as climatic demands, partially accounted for the effect of harmony and affective autonomy on alcohol consumption. This research highlights that cultural values, and their interaction with gender, should be an important consideration for international public health organizations aiming to tackle the problems associated with alcohol consumption, but that future research is required to fully understand the link between cultural values and alcohol.

## Introduction

Alcohol consumption is known to have a range of negative consequences at the individual and societal levels. In 2012, alcohol consumption was estimated to have caused 3.3 million deaths globally, a figure that corresponds to 5.9% of all deaths that year (World Health Organization [WHO], 2014a). It is strongly associated with hypertension, liver cirrhosis, and chronic pancreatitis (Corrao et al., 2004), and causes an economic burden of around 1% on Gross Domestic Product (Sassi, 2015). An effective attempt to prevent and reduce harmful alcohol consumption requires an understanding of the complex network of risk factors that cause it (Miranda et al., 2008). Since many of these risk factors are likely to be affected by cultural factors (e.g., Neumark et al., 2003; Chartier et al., 2013), or to be at the cultural level, a better understanding of this complexity necessitates an examination of their socio-cultural underpinnings.

Past research has identified a range of correlates and predictors of alcohol consumption including attitudes, subjective norms, and self-efficacy (Cooke et al., 2016), marital and employment status (Temple et al., 1991), and various demographic factors (Moore et al., 2005). In general, most conducted studies have focused on correlates and predictors uniquely at the individual level. Shifting this focus to examining predictors at a macro level (country, regional or cultural; Bronfenbrenner, 1974) should help facilitate an understanding of the overall pattern of correlates and predictors of alcohol consumption and potentially aid in the development of large scale interventions led by international organizations such as the World Health Organization [WHO] (2014a). In other words, the effectiveness of an intervention targeting harmful alcohol consumption may be improved with a better understanding of the cultural factors that are associated with this risky behavior. One such country-level factor is cultural values – the degree to which members of a cultural group value autonomy/individualism vs. embeddedness/collectivism, for example. Past research, which has shown differences in alcohol consumption based on within-country cultural differences, has highlighted the need for more research into cultural risk-factors (Chartier et al., 2013). However, to the best of our knowledge, the associations between cultural values and alcohol consumption have not been systematically investigated.

**FIGURE 1:**
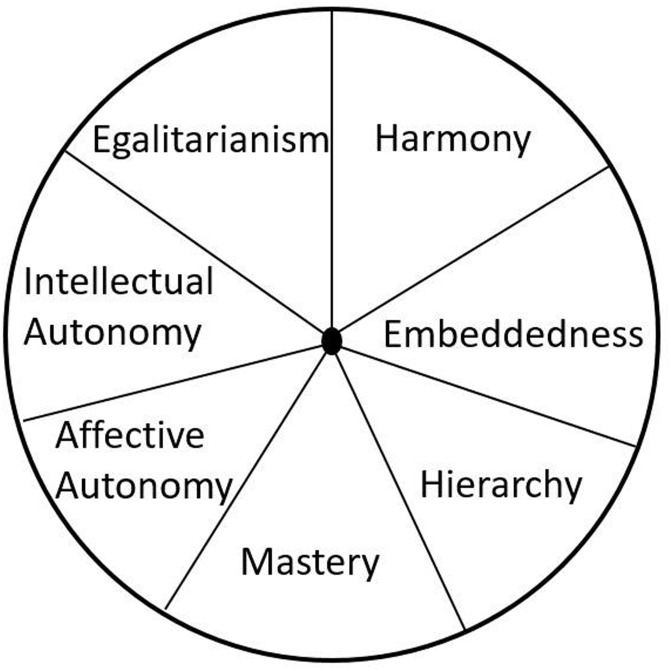
Schwartz’ cultural value orientation model.

The theoretical model we focus on in this study is Schwartz’s model of Cultural Value Orientation (Schwartz, 2006), which is derived from his prior theory of human values (Schwartz, 1992) and related to other influential models of cultural values such as Hofstede’s (1980) cultural dimensions and Inglehart (1977) modernization theory (e.g., Dobewall and Rudnev, 2014). Schwartz posits seven *a priori* cultural value orientations that correspond to cultural ideals, which are shared conceptions of good and desirable cultural standards (Schwartz, 2006, p. 139). The seven cultural value orientations are Intellectual Autonomy (being independent), Affective Autonomy (pursuing positive affective experiences), Mastery (encouraging self-assertion), Hierarchy (unequal distribution of power), Embeddedness (being part of a collective), Harmony (being at ease with the world), and Egalitarianism (being concerned for others). The seven cultural value orientations can be ordered along three dimensions: Embeddedness vs. Autonomy, Hierarchy vs. Egalitarianism, and Mastery vs. Harmony (see **Figure 1**). A crucial aspect of this model is that the cultural value orientations are ordered in a systematic way according to their motivational synergies and conflicts. Schwartz (1992, 2006) proposed a circumplex arrangement, making specific predictions about the pattern of relations between values. Specifically, the motivational synergies and conflicts in the model predict that the importance ratings of adjacent cultural value orientations are positively correlated with each other, orthogonal cultural value orientations are relatively weakly or unrelated to each other, and opposing cultural value orientations are negatively correlated. For example, if a variable (e.g., alcohol consumption) is positively related with Intellectual Autonomy, this variable is also expected to be positively related to Egalitarianism and Affective Autonomy, but negatively related with Embeddedness.

Several studies have found positive associations between Autonomy and a wide range of country-level variables which are usually considered as beneficial to society. These include peacefulness (Basabe and Valencia, 2007), domestic product per capita, democratization, gender equality (Schwartz, 2006), and well-being (Diener et al., 1995). In line with predictions made by Schwartz, the same variables were negatively related to Embeddedness. However, high levels of Autonomy also result in less commitment to norms and traditions (Schwartz, 2006). These findings allow us to derive specific hypotheses regarding the relations between Autonomy and Embeddedness with alcohol consumption.

Alcohol consumption is often associated with socially undesirable behaviors and health problems (World Health Organization [WHO], 2014a), both of which are threatening to the collective and tradition. Further, many religions that totally forbid alcohol consumption, such as Islam, promote cultural embeddedness (Schwartz, 2009). It therefore seems reasonable to believe that alcohol consumption would be negatively associated with embeddedness and related values (respect for tradition, obedience, social order), and because of the underlying motivational continuum of Schwartz’s (2006) cultural value orientations, positively related to Autonomy.

Additionally, the rights of women in many countries are more limited than the rights of men, a case which is especially true in countries with lower Intellectual Autonomy (Schwartz, 2006). Many cultures and societies also hold negative attitudes toward women drinking alcohol, but accept this behavior in men (Pretorious et al., 2009). We were therefore also interested in assessing whether there were differences in the alcohol consumption of males and females based on cultural values.

With the above considered, the aim of our study was to investigate the relationship between cultural value orientations, especially Autonomy and Embeddedness, and alcohol consumption. Based on previous findings (Martin and Sher, 1994; Perkins, 2002) we expected Autonomy to be positively associated with alcohol consumption, and Embeddedness to be negatively related with alcohol consumption. Because of the circumplex structure of Schwartz’s CVO-model, we also expected positive, albeit weaker, correlations between Egalitarianism and alcohol consumption and also negative associations with Hierarchy, because Egalitarianism and Hierarchy are motivationally similar to Autonomy and Embeddedness, respectively (Schwartz, 2006).

Finally, beyond simply understanding whether Cultural Values Orientations and encultured alcohol consumption share an association, we were also interested in understanding whether any observed association might be explained by ecological variables that covary with a country’s geographical position. The prevalence of non-zoonotic parasitic diseases such as cholera, which are more highly present in southern countries compared to northern countries, have been found to be positively associated with collectivist cultural values and negatively correlated with democracy and human freedom (Thornhill et al., 2010). Climate (temperate vs. demanding) has also been shown to be a critical predictor of a range of cultural variables, such as freedoms of press and freedom of discrimination. Van de Vliert (2013), for example, demonstrated that freedom of expression was worse in poor countries with demanding climate, best in rich countries with a demanding climate, and intermediate in countries with temperate climates.

## Materials and Methods

### Data Sources

The values we used for cultural value orientations were those obtained by Schwartz between 1988 and 2000 (please refer to Schwartz, 2006, for full details). Specifically, we used the country averages of all seven cultural value orientations which Schwartz (2008) made openly available. Where cultural value orientations were presented for different cultural groups within a single country (e.g., East and West Germany), these values were averaged. To be clear, what this means is that for the sake of this article a culture refers to the average culture measured within a nation. Naturally, within a nation there are usually many different cultural groups, but we shall consider a nation/country as a single cultural unit. In total, the cultural value orientations for 74 nations were included in the analyses.

We obtained our estimates of total alcohol consumption per capita (15+ years; liters of pure alcohol per year) for these countries from the World Health Organization, Global Health Observatory data repository, which is available online (World Health Organization [WHO], 2017). A 3-year average value is available for males and females separately for the period 2008–2010. Year specific values are available for all years for the total adult population. We also included estimates for Income (Gross National Income per capita, based on purchasing power parity; data are in current international dollars based on the 2011 ICP round) and for Education (average years of education by people ages 25 and older) to act as control variables. The decision to control for these two variables was empirically driven because they have been found to be associated with alcohol consumption (Grittner et al., 2013; Barr et al., 2016). These values are available for individual years and were extracted from the World Bank Open Data set (The World Bank Group [WBG], 2016) and Human Development Index Data set (United Nations Developmental Programme, 2014), respectively. The average country latitudes were obtained from a public data set which can be retried at https://developers.google.com/public-data/docs/canonical/countries_csv.

### Statistical Analysis

Prior to analysis missing data were imputed using Multiple Imputation by Chained Equations (Raghunathan et al., 2001). We then evaluated the relationship between cultural value orientations and alcohol consumption in four stages:

First we conducted partial correlations between cultural value orientations and alcohol consumption for males, females, and total population (2008–2010 period) controlling for the effect of the mean Income and Education for these years. For significance tests, we applied a Holm′s adjustment to correct for multiple comparisons. We interpret the effect size of correlation coefficients using the guidelines described by Cohen (1988).

Secondly, in line with what might be expected from a circumplex model, VIF analyses revealed significant multicollinearity between cultural value orientations. We therefore used linear ridge regression, a technique that uses a shrinkage constant (*k*) to make regression coefficients more stable in the case of multicollinearity (Ryan, 2008), to determine the amount of unique variance in alcohol consumption that can be accounted for by cultural value orientations. One effect of adding a shrinkage parameter to regression equations is that the estimates of effect size are conservative (Draper and Smith, 1988). We included the same variables in the model as we did in the partial correlations with the addition of the dummy variable Gender and seven Gender-by- cultural value orientation interaction terms.

To assess the sensitivity of our findings we conducted two further sets of partial correlations. Partial correlations were calculated for alcohol consumption and cultural value orientations for three time periods (1990, 2000, and 2010). The effects of Income and Education for the corresponding years were controlled for. Partial correlations for males, females and total sample (once more for the period 2008–2010) were then calculated, but for two subsamples, one containing only European countries (*n* = 35), the other containing the remainder non-European countries (*n* = 39). The effects of mean Income and Education for the corresponding years, and corresponding countries, were controlled for.

As will become apparent, our results revealed that Cultural Value Orientations were associated with alcohol consumption across the 74 nations. Our final stage of analysis was an assessment of whether latitude – and by extension a range of ecological factors that have been shown to covary with latitude – might account for the variance between values and alcohol consumption. To do this, 14 statistical mediation analyses were conducted following the proposals of Baron and Kenny (1986). For each analysis, the effect of a Cultural Value Orientation on alcohol consumption (male or female) via the mediating effect of latitude was assessed using a series of regression analyses. To conclude that a latitude is a mediator, the effect between the cultural value and latitude must be significant (path a); the effect between latitude and alcohol consumption must be significant (path b); the total effect of the cultural value on alcohol consumption must be significant (path c); and the effect of the cultural value on alcohol consumption after adjusting for the mediator (path c′) should be smaller than that for path c. We assessed any indirect effects via an inspection of bootstrapped confidence intervals.

## Results

### Descriptive Statistics

Based on World Health Organization defined geographical regions our sample comprised 9 countries from Africa, 12 from the Americas, 4 from the Eastern Mediterranean Region, 35 from Europe, 5 from the South East Asia Region and 9 from the Western Pacific Region. The average Income (GNI) for the period 2008–2010 was $1.08 trillion. For the same years, the mean years of schooling (Education) for countries in our sample was 9.41 years (*Range*: 2.23 – 12.90). In total, males were shown to consume more liters of pure alcohol per year (*M* = 12.97, *Range*: 0.40 – 23.90) than women (*M* = 4.32, *Range*: 0.00 – 7.80). In terms of cultural value orientations, Egalitarianism was the most highly valued across all countries (*M* = 4.68) while Hierarchy was the least (*M* = 2.35).

### Partial Correlations

**Table 1** presents the partial correlations between cultural value orientations and estimates of alcohol consumption for males and females. Inspection of these profiles reveals a similar general pattern for males and females, although some striking differences are evident.

For males there was a significant positive correlation between Harmony and alcohol consumption, and a significant negative correlation between Embeddedness and alcohol consumption. There were weak, and not significant, positive correlations between alcohol consumption and the values of Autonomy. There were also weak negative correlations between Hierarchy, Mastery and Egalitarianism, and alcohol consumption, but none of these were significant.

For females it is evident that the overall pattern of associations was stronger than for males. There were medium-sized, significant positive correlations between Autonomy (Intellectual and Affective) and Harmony with alcohol consumption, and medium-sized, significant negative correlations between Embeddedness and Hierarchy with alcohol consumption. Egalitarianism and Mastery were not significantly correlated with alcohol consumption for females.

**Table 1:** Partial correlations between alcohol, recorded per capita (15+ years) consumption (in liters of pure alcohol) and CVOs in the period 2008–2010.

	Cultural value						
**Gender**	**Harm.**	**Embed.**	**Hier.**	**Mast.**	**Aff. Auton.**	**Intel. Auton.**	**Egal.**

Males	0.33^∗∗^	-0.26^∗^	-0.20	-0.02	0.16	0.16	-0.02
Females	0.32^∗∗^	-0.45^∗∗∗^	-0.31^∗∗^	0.04	0.27^∗^	0.33^∗∗^	0.23
Total	0.40^∗∗^	-0.38^∗∗^	-0.29^∗^	-0.05	0.26^∗^	0.23	0.18

*CVOs, cultural value orientations; Harm., harmony; Emb., embeddedness; Hier., hierarchy; Mast., mastery; Aff. Auton., affective autonomy; Intel. Auton., intellectual autonomy; Egal., egalitarianism. Mean GNI and years of schooling for 2008–2010 were controlled for (*N* = 74). ^∗^*p* < 0.05, ^∗∗^*p* < 0.01, ^∗∗∗^*p* < 0.001.*

### Ridge Regression

The linear ridge regression model supported the above observations (**Table 2**).

**Table 2:** Ridge regression model for alcohol consumption in males and females.

Variable	*R*^2^	Adjusted *R*^2^	*F*	β	*SE*(β)^a^	*F*
	0.647	0.598	11.908^∗∗∗^			
Income				0.021	0.017	1.613
Education				0.103	0.017	37.518^∗∗∗^
Gender				0.075	0.005	274.889^∗∗∗^
Harm.				0.047	0.019	6.134^∗^
Emb.				-0.077	0.012	43.481^∗∗∗^
Hier.				-0.046	0.017	7.157^∗∗^
Mast.				0.006	0.019	0.098
Aff.Auton.				0.047	0.013	13.059^∗∗∗^
Intel.Auton.				0.040	0.013	10.078^∗∗^
Egal.				-0.032	0.018	3.037
Gender × Harm.				0.081	0.005	271.603^∗∗∗^
Gender × Emb.				0.061	0.008	52.826^∗∗∗^
Gender × Hier.				0.060	0.010	36.578^∗∗∗^
Gender × Mast.				0.075	0.005	261.381^∗∗∗^
Gender × Aff.Auton				0.088	0.005	312.207^∗∗∗^
Gender × Intel.Auton.				0.082	0.004	427.272^∗∗∗^
Gender × Egal				0.070	0.005	238.674^∗∗∗^

*^∗∗∗^*p* < 0.001, ^∗∗^*p* < 0.01, ^∗^*p* < 0.05. ^a^Bootstrap (1000) estimate of SE, Shrinkage parameter (*k*) = 2.0, Harm, harmony; Emb., embeddedness; Hier., hierarchy; Mast., mastery; Aff. Auton., affective autonomy; Intel. Auton., intellectual autonomy; Egal., egalitarianism.*

The overall model accounted for a large proportion of variance in alcohol consumption, *r*^2^ = 0.65, Adjusted *r*^2^ = 0.60, *F*(17,130) = 11.91, *p* < 0.001. Of the control variables, Education, and Gender were found to be significantly and positively associated with alcohol consumption. Income was not significantly associated with alcohol consumption. A number of cultural value orientations accounted for unique variance in the model. Affective and Intellectual Autonomy, and Harmony, were significantly positively associated with alcohol consumption. Embeddedness and Hierarchy were significantly negatively associated with alcohol consumption. Mastery and Egalitarianism showed no significant associated with consumption. All seven interaction terms were found to be significant.

**FIGURE 2:**
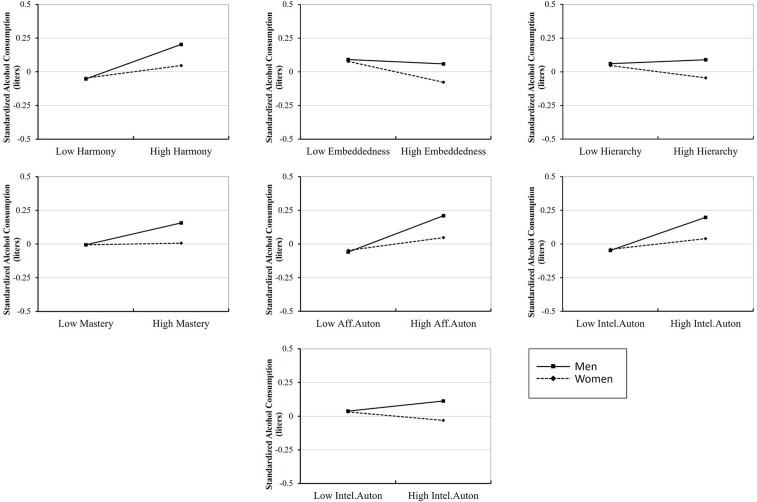
Gender-by-cultural value orientation interaction plots for the seven cultural values. The *y*-axis for each graph represents alcohol consumption in terms of standard deviations away from the mean (*M* = 0).

**Figure 2** shows the significant gender-by-cultural value interactions plotted on an axis representing standardized alcohol consumption. What these plots therefore show is that when all else is equal, for countries low in all seven values males consume roughly the average level of alcohol for males, and females consume roughly the average levels of alcohol for females. The interactions were apparent when observing gender differences in countries with high levels of each value. For Harmony, Mastery, and Affective and Intellectual Autonomy, in countries with high levels of these values females drank around the average level of alcohol for females (although there was a small increase in consumption), but males drank roughly 0.25 standard deviations more than the mean per year. In contrast, for Embeddedness and Hierarchy, when all else was held equal, males drank similar levels of alcohol in countries with low and high levels of these values, but females drank less alcohol in countries with high levels of these values. For Egalitarianism, compared to countries with low levels of this value, males drank more alcohol in countries with high Egalitarianism, and females drank slightly less alcohol in countries with high Egalitarianism.

### The Association between Cultural Value Orientations and Alcohol Consumption Over Time and within European and Non-european Subsamples

Inspection of **Table 3** reveals that the patterns of associations between cultural value orientations and alcohol consumption at three time points were similar, although over time the strength of associations was weakening. Furthermore, these patterns are consistent with the overall pattern of associations shown in **Table 1**.

**Table 3:** Partial correlations between alcohol, recorded per capita (15+ years) consumption (in liters of pure alcohol) and CVOs across 20 years.

	Cultural value						
**Year**	**Harm.**	**Embed.**	**Hier.**	**Mast.**	**Aff. Auton.**	**Intel. Auton.**	**Egal.**

1990	0.44^∗∗∗^	-0.48^∗∗∗^	-0.40^∗∗^	-0.14	0.36^∗∗^	0.41^∗∗∗^	0.34^∗∗^
2000	0.43^∗∗^	-0.49^∗∗∗^	-0.38^∗∗^	-0.10	0.37^∗∗^	0.41^∗∗∗^	0.37^∗∗^
2010	0.40^∗∗^	-0.38^∗∗^	-0.29^∗^	-0.05	0.26^∗^	0.23	0.18

*CVOs, cultural value orientations; Harm., harmony; Emb., embeddedness; Hier., hierarchy; Mast., mastery; Aff. Auton., affective autonomy; Intel. Auton., intellectual Autonomy; Egal., egalitarianism. GNI and mean years of schooling for the corresponding years were controlled for. ^∗^*p* < 0.05, ^∗∗^*p* < 0.01, ^∗∗∗^*p* < 0.001.*

**Table 4** presents Pearson correlations for males and females (2008–2010 values) for two subsets; 35 European nations and 39 non-European nations. The general pattern of associations between alcohol consumption and cultural values was similar between the subsamples, and to the results presented in **Table 1**. This was particularly the case for Harmony (medium positive), Embeddedness (medium negative), Mastery (negligible) and Affective Autonomy (weak positive). For Hierarchy, European countries showed a weaker negative correlation (*r* = -0.07) compared to non-European countries (*r* = -0.23). For Intellectual Autonomy, European countries showed a stronger, but still weak, positive correlation (*r* = 0.16) than non-European countries (*r* = 0.08), and for Egalitarianism European countries had a medium positive correlation (*r* = 0.30), while non-European countries showed little evidence of any association (*r* = 0.02).

**Table 4:** Partial correlations between alcohol, recorded per capita (15+ years) consumption (in liters of pure alcohol) and CVOs in the period 2008–2010 for European countries (*n* = 35) and non-European countries (*n* = 39).

	Cultural value						
**Gender**	**Harm.**	**Embed.**	**Hier.**	**Mast.**	**Aff. Auton.**	**Intel. Auton.**	**Egal.**

**European**							
Males	0.34	-0.46^∗^	-0.05	0.11	0.20	0.15	0.28
Females	0.20	-0.53^∗∗^	-0.15	0.18	0.14	0.27	0.37^∗^
Total	0.36^∗^	-0.48^∗∗^	-0.07	0.08	0.18	0.16	0.30
**Non-European**							
Males	0.07	0.07	-0.09	-0.03	-0.07	-0.08	-0.42^∗^
Females	0.24	-0.32	-0.28	0.07	0.30	0.23	0.04
Total	0.20	-0.21	-0.23	0.02	0.19	0.08	0.02


*CVOs, cultural value orientations; Harm., harmony; Emb., embeddedness; Hier., hierarchy; Mast., mastery; Aff. Auton., affective autonomy; Intel. Auton., intellectual autonomy; Egal., egalitarianism. Mean GNI and years of schooling for 2008–2010 were controlled for. ^∗^*p* < 0.05, ^∗∗^*p* < 0.01, ^∗∗∗^*p* < 0.001.*

Some clear differences were observed when inspecting consumption for males and females separately. Firstly, while males in the European subsample had similar correlations to those shown in **Table 1**, males in the non-European subsample showed very little association between alcohol consumption and the cultural values. The exception was for Egalitarianism, which showed a moderate negative correlation (*r* = -0.42) which differed from the European subsample, where males showed a positive correlation (*r* = 0.28). For females the correlations were remarkably similar for European and non-European subsamples, although an exception was Egalitarianism, which showed little association with alcohol consumption in non-European countries (*r* = 0.04), but a medium positive correlation in European countries (*r* = 0.37).

### Latitude as a Mediator of the Relationship between Cultural Value Orientations and Alcohol Consumption

As a primary step to understanding the relationship between latitude and alcohol consumption, Pearson’s correlations were calculated between latitude and male and female alcohol consumption. Significant positive correlations were found for male, *r* = 0.35, *p* = 0.002, and female alcohol consumption, *r* = 0.37, *p* = 0.001. Thus there appears to be evidence of a north–south consumption gradient.

The complete output from the 14 statistical mediation analyses are presented in Supplementary Tables S1–S7. Mastery and Egalitarianism did not present all the significant effects, as defined by Baron and Kenny (1986), for the effect of these values on alcohol consumption to be mediated by latitude. In all cases, the Sobel test did not indicate a significant statistical mediation effect. However, for Harmony (*c*′_male_ = 0.095, [CI 0.022, 0.217]; *c*′_female_ = 0.081, CI [0.011, 0.200]), Hierarchy in males (*c*′_male_ = -0.094, [CI -0.216, -0.011]), and Affective Autonomy (*c*′_male_ = 0.077, [CI 0.006, 0.213]; *c*′_female_ = 0.066, CI [0.001, 0.192]), and inspection of the 95% confidence intervals suggested that latitude was able to account for a partial but meaningful amount of the effect of these values on alcohol consumption. Embeddedness (*c*′_male_ = -0.072, [CI -0.199, 0.009]; *c*′_female_ = -0.049, CI [-0.167, 0.011]), and Intellectual Autonomy (*c*′_male_ = 0.098, [CI -0.019, 0.246]; *c*′_female_ = 0.047, CI [-0.055, 0.178]), and Hierarchy for females only (*c*′_female_ = -0.069, CI [-0.189, 0.001]) did not show evidence of meaningful partial mediation effects.

## Discussion

Our analyses demonstrate that there is a pattern with which cultural value orientations are associated with alcohol consumption. There was a clear association between the Autonomy vs. Embeddedness value dimension and levels of consumption. For males and females, countries which highly valued Intellectual and Affective Autonomy (Germany and the United Kingdom for example) were generally associated with higher levels of consumption, and countries which highly valued Embeddedness (such as Yemen and Senegal) were generally associated with lower levels of consumption. This pattern of associations was found to be robust over three time points, and reasonably similar across subsamples of European and non-European nations. These data therefore suggest that certain cultural characteristics are associated with a propensity for higher cultural alcohol consumption, a behavior associated at the individual level with hypertension, liver cirrhosis, and chronic pancreatitis (Corrao et al., 2004) and considered as a modifiable behavioral risk factor for non-communicable diseases (NCDs) including cardiovascular disease, cancer, chronic respiratory disease and diabetes, and at a societal level a causes of social and economic losses (World Health Organization [WHO], 2014b).

We also assessed the possibility that country latitude, and by extension covariates of latitude such as heat demands and presence of parasitic diseases, might offer some explanation for the observed relationship between Cultural Value Orientations and alcohol consumption. Latitude was shown to be a weak but meaningful partial mediator of the relationship with alcohol consumption for Harmony, Affective Autonomy, and Hierarchy (males only for this value), but not Intellectual Autonomy or Embeddedness. Since this means that latitude and its associated variables should be considered as an incomplete explanation for how Cultural Value Orientations account for national differences in alcohol consumption, it is necessary to consider which other variables may also be accountable.

A sociocultural approach to describing alcohol-related behaviors has been to categorize cultures based on clusters of traits (Room and Mäkelä, 2000). One category to emerge from this approach contains what might be referred to as abstinent societies. These societies, including some Islamic and Hindu societies, are characterized by an absolute religious and legal forbiddance of alcohol consumption. The association between religiousness and alcohol consumption is well established (e.g., Haber and Jacob, 2007; Neighbors et al., 2013) and specific religious beliefs, such as that homosexuality, abortion and divorce are bad, have also been found to be negatively correlated with the cultural value Harmony (Schwartz, 2006). Thus one alternative to ecological variables that remains to be tested is that there may be a relationship between cultural values, religion, and alcohol consumption.

Similar research questions might also emerge for other cultural variables. Populations of different countries, for varying political and historical reasons, experience differing degrees of freedom, equality, relative safety, and social control that are likely to provide environments in which there are more or less opportunities to consume alcohol. Many of such variables are likely to directly explain some partial variance in the cultural alcohol consumption patterns. In support of this claim, American states that have become more politically liberal over time have increased their alcohol consumption (Yakovlev and Guessford, 2013). Naturally, such variables are also likely to be correlated with cultural values. For example, gender equality has been shown to be strongly positively correlated with Intellectual Autonomy (Schwartz, 2006). Autonomous countries have also been shown to enjoy fewer strict social rules (Basabe and Valencia, 2007). Thus, one future line of investigation should be examining the interaction between variables such as political liberalism, cultural values of autonomy, and alcohol consumption.

### The Case of Harmony

The finding that Harmony was positively associated with alcohol consumption needs further explanation because the neighboring cultural value orientation, Embeddedness, was negatively correlated with it. Based on the motivational synergies proposed by Schwartz, we implicitly expected Harmony to be unrelated or slightly negatively related to alcohol consumption because the underlying motivational synergies are related to Embeddedness, which was, as predicted, negatively correlated with alcohol consumption. We believe that this can be explained with the definition of Harmony that “emphasizes fitting into the world as it is, trying to understand and appreciate rather than to change” (Schwartz, 2006, p. 141). In line with this, social motives related to Harmony such as having a good time with friends have been found to be predictors of alcohol consumption (Kuntsche et al., 2005). Furthermore, research that has looked into the relationship between cultural value orientations and peacefulness related variables, such as violent inequality, has also found that the sign of the correlation coefficients was reversed for Embeddedness and Harmony (Basabe and Valencia, 2007). This suggests that the underlying motivation for at least those two adjacent cultural value orientations can also be conflicting in relation to specific external variables.

### The Effect of Gender

Our results indicated some interesting gender effects. Firstly, the partial correlations between cultural values and alcohol consumption were stronger for women than for men, suggesting that cultural values are a more reliable predictor of alcohol consumption in women than for men. Secondly, the gender-by-cultural value interactions were all found to be significant. These indicated that alcohol consumption in women appeared to differ only slightly, all else being equal, between countries with high and low values of each value. Nonetheless, despite the small changes in consumption, it was evident that female consumption was higher in countries that highly valued Autonomy, and lower in countries that highly valued Embeddedness. Men tended to consume more alcohol in countries with higher levels of each value, the exceptions being Hierarchy and Embeddedness, for which the differences were small. These findings are in line with what is expected from opposing cultural values and supports the relevance of the Autonomy-Embeddedness dimension for determining cultural alcohol consumption (the Harmony-Mastery and Egalitarianism-Hierarchy dimensions showed similar patterns of results for each of the values). They also suggest that Embeddedness and its adjacent value Hierarchy, which are more highly associated with less gender equality (Schwartz, 2006) and negative attitudes about women drinking alcohol (Pretorious et al., 2009), are relevant cultural values to consider in accounting for gender differences in alcohol consumption.

### Implications for Public Health

A broad aim of international organizations concerned with public health is to collect and use data to inform national governments on how best to tackle health issues. In relation to alcohol, the WHO in particular has made a continued effort to collect information and to use this to help nations reduce the harmful use of alcohol and its consequences (World Health Organization [WHO], 2014a). Although the WHO uses considerable amounts of information to help with this process, we argue that cultural information such as a country’s value profile may well serve as a useful tool for helping to inform policy. At a basic level, our results suggest that the WHO should prioritize tackling alcohol consumption in countries that are high in Autonomy and Harmony, and low in Embeddedness and Hierarchy. At the very least, our results indicate that future research should be directed at further understanding the causal mechanisms between cultural values and alcohol consumption, and their interaction with gender, the results of which could be used to tailor intervention programs that are culturally sensitive.

Importantly, alcohol consumption is just one of four modifiable behavioral risk factors of NCDs, which are responsible for 70% of global deaths (World Health Organization [WHO], 2014b). The other risk factors are smoking, physical inactivity, and diet. Having shed some light on the cultural underpinnings of alcohol consumption, an obvious next step is to create similar profiles and models to understand the cultural underpinnings of these other risk factors. When combined, this information may prove to be highly impactful on how governments opt to implement health policies aimed at reducing the prevalence of NCDs.

### Strengths

The results of our study are interesting because our variables – cultural value orientations, Education, Income and Alcohol consumption– were measured independently and are based on different sources (self-report vs. official statistics). This rules methodological bias out as a hidden moderator – a problem which many studies that rely on self-reports face (Richardson et al., 2009). Also, the data we used is highly reliable and stable, given that it relies on around 40,000 participants, whose responses were used to estimate the 74 country averages of the cultural value orientations.

A test of the validity revealed a similar overall CVO profile for three different time points over 20 years and for the European subset of countries for men and women for the time period 2008–2010: These findings suggest firstly that the identified pattern of associations between cultural value orientations and alcohol is not specific to a single time point, although it appears that the overall association between cultural values and alcohol is decreasing: likely a result of converging levels of alcohol consumption across countries (Room and Mäkelä, 2000). This is interesting since the global trend and current projections for 2025 are an increase in alcohol consumption (World Health Organization [WHO], 2014a). At a regional level, however, there are some differences, with Europe showing a trend for decreases in consumption.

The pattern of associations was replicated with a European subsample of our data. Inspecting the total levels of alcohol consumption, the pattern of correlations between the European sample and the total sample (**Table 1**) were, with the exception of Hierarchy, remarkably similar. Hierarchy had a weaker negative correlation in the European sample than the total sample. The non-European sample was also fairly similar to the total sample, particularly for Harmony, Embeddedness, Hierarchy, Mastery and Affective Autonomy.

### Limitations

In our analyses we controlled for two variables that have been shown to be associated with alcohol consumption; income and education (Grittner et al., 2013; Barr et al., 2016). Of course, other variables such as the average age of a population, the price of alcohol, national alcohol laws/policies, degree of urbanization etc. also influence alcohol consumption and were not controlled for. These variables should be considered in future research. Since the number of predictors/variables determines the sample size required for sufficiently powered statistical analyses, including additional covariates in future research will require multi-level data from a larger sample of countries. Further, although not reported, our analyses revealed that cultural value orientations were correlated with income and education. Thus it is likely that our analysis controlled out some of the cultural variance in alcohol consumption.

When examining the association between cultural value orientations and alcohol consumption at different years we used the same CVO values and so it might be argued that our results do not entirely accurately reflect the time specific associations. However, cultural value orientations are claimed to be stable over time (Schwartz et al., 2000; Hofstede, 2001; Manfredo et al., 2017; Tormos et al., 2017) and this suggests that over a relatively short period of time using the same values of cultural value orientations is legitimate.

While the associations between total alcohol consumption and values for European and non-European countries were similar for Harmony, Embeddedness, Mastery and Affective Autonomy, some notable differences were evident between the European subsample and non-European subsample. Firstly, with the exception of Egalitarianism, alcohol consumption in males from non-European countries showed little evidence of being correlated with cultural value orientations. In this sense, the pattern of associations for males displayed in **Table 1** can be said to be driven by European countries. In other words, while encultured drinking levels by men in Europe is associated with cultural values, outside of Europe these values are unrelated to consumption. Why this might be, lies beyond the scope of this discussion. For women, the associations between consumption and values were, more or less, consistent between the subsamples, although of note was that Egalitarianism was more strongly associated with alcohol consumption in European countries than non-European countries. This highlights the complexity of the relationship between values and alcohol consumption, specifically demonstrating that beyond the presented two-way gender-by-value interactions, there may also be three-way region-by-gender-by-value interactions. Future research, incorporating larger data-sets should consider this complex interplay of variables.

### Future Research

In addition to investigating the interplay between cultural values, alcohol consumption, and variables such as religiosity, we suggest that future research should look into causal relations between cultural value orientations and alcohol consumption, using a multi-level longitudinal design. Prior pseudo-longitudinal work using cultural value orientations to predict socioeconomic development and democratization has found that cultural value orientations predict said national variable rather than vice versa (Schwartz, 2006). Also, a longitudinal study has found that values and value-expressive behaviors influence each other reciprocally, but values tended to have a stronger influence on behavior then behavior on values (Vecchione et al., 2015). We are therefore expecting that cultural value orientations influence alcohol consumption directly, but that alcohol consumption can also influence cultural value orientations through indirect routes such as conflict and increased levels of psychopathology: both values and alcohol consumption have been found to be related to or causing aggression and depression (Hoaken and Stewart, 2003; Boden and Fergusson, 2011; Hanel and Wolfradt, 2016; Souchon et al., submitted). A multi-level design would allow us to test whether the same pattern observed on a country level can also be found on an individual level consistently in each country.

## Conclusion

Overall, our findings highlight new important predictors of encultured alcohol consumption across 74 countries, representing around 90% of the world’s population. The association between Cultural Value Orientations and alcohol consumption was stronger for women than for men. Both of these findings can be of value to international organizations that aim to tackle public health issues, such as the negative effects of excessive drinking. Finally, latitude, and by extension other ecological covariates of latitude such as climatic demands, were shown to partially explain the relationship between Cultural Value Orientations and alcohol consumption, but the weak indirect effects and inability to account for the relationship between Embeddedness, Intellectual Autonomy, and Hierarchy (females only) and alcohol consumption indicates that future research should be directed at investigating this relationship further.

## Author Contributions

RI: Conducted data analysis and was involved with writing the first draft. Later revisions were also made by RI. SdS: Was involved with the conceptualization of the paper and with making revisions to the manuscript. RB: Was involved with obtaining data and with making revisions to the manuscript. PH: Was involved with the conceptualization of the paper, obtaining the data, writing the first draft, and making revisions to the manuscript.

## Conflict of Interest Statement

The authors declare that the research was conducted in the absence of any commercial or financial relationships that could be construed as a potential conflict of interest.
