# Supplementary material for: Cultural Value Orientations and Alcohol Consumption in 74 Countries: A Societal-Level Analysis
Source: Front Psychol. 2017 Nov 20;8:1963. doi: 10.3389/fpsyg.2017.01963 (PMC5702438; doi:10.3389/fpsyg.2017.01963)
Supplement: Supplementary file 1 [file Table_1.DOCX]

| Table S1.  *Mediation Analyses for the association between Harmony and Alcohol Consumption in males and females.* | | | | |
| --- | --- | --- | --- | --- |
| Variable | R^2^ | *F* | β | *p* |
| 1. *Latitude* | .13 | 10.40 |  |  |
| Harmony |  |  | .36 | .002 |
| 1. *Alcohol Male* | .12 | 9.99 |  |  |
| Latitude |  |  | .35 | .002 |
| 1. *Alcohol Male* | .10 | 8.31 |  |  |
| Harmony |  |  | .32 | .005 |
| *c’. Alcohol Male* | .17 | 7.09 |  |  |
| Harmony |  |  | .23 | .054 |
| Latitude |  |  | .27 | .023 |
| Sobel Test = .09, *SE* = .05, *p* = .07 | | | | |
| 1. *Latitude* | .13 | 10.40 |  |  |
| Harmony |  |  | .36 | .002 |
| 1. *Alcohol Female* | .14 | 11.24 |  |  |
| Latitude |  |  | .37 | .001 |
| 1. *Alcohol Female* | .22 | 20.80 |  |  |
| Harmony |  |  | .47 | <.001 |
| *c’. Alcohol Female* | .27 | 13.10 |  |  |
| Harmony |  |  | .39 | .001 |
| Latitude |  |  | .23 | .039 |
| Sobel Test = .08, *SE* = .05, *p* = .09 | | | | |
