# Supplementary material for: Cultural Value Orientations and Alcohol Consumption in 74 Countries: A Societal-Level Analysis
Source: Front Psychol. 2017 Nov 20;8:1963. doi: 10.3389/fpsyg.2017.01963 (PMC5702438; doi:10.3389/fpsyg.2017.01963)
Supplement: Supplementary file 2 [file Table_2.DOCX]

| Table S2.  *Mediation Analyses for the association between Embeddedness and Alcohol Consumption in males and females.* | | | | |
| --- | --- | --- | --- | --- |
| Variable | R^2^ | *F* | β | *p* |
| 1. *Latitude* | .15 | 13.07 |  |  |
| Embeddedness |  |  | -.39 | <.001 |
| 1. *Alcohol Male* | .12 | 9.99 |  |  |
| Latitude |  |  | .35 | .002 |
| 1. *Alcohol Male* | .25 | 13.41 |  |  |
| Embeddedness |  |  | -.50 | <.001 |
| *c’. Alcohol Male* | .27 | 13.38 |  |  |
| Embeddedness |  |  | -.42 | <.001 |
| Latitude |  |  | .18 | .101 |
| Sobel Test = -.07, *SE* = .05, *p* = .14 | | | | |
| 1. *Latitude* | .15 | 13.07 |  |  |
| Embeddedness |  |  | -.39 | <.001 |
| 1. *Alcohol Female* | .14 | 11.24 |  |  |
| Latitude |  |  | .37 | .001 |
| 1. *Alcohol Female* | .45 | 57.78 |  |  |
| Embeddedness |  |  | -.67 | <.001 |
| *c’. Alcohol Female* | .46 | 30.05 |  |  |
| Embeddedness |  |  | -.62 | <.001 |
| Latitude |  |  | .13 | .192 |
| Sobel Test = .05, *SE* = .04, *p* = .23 | | | | |
