# Supplementary material for: Cultural Value Orientations and Alcohol Consumption in 74 Countries: A Societal-Level Analysis
Source: Front Psychol. 2017 Nov 20;8:1963. doi: 10.3389/fpsyg.2017.01963 (PMC5702438; doi:10.3389/fpsyg.2017.01963)
Supplement: Supplementary file 3 [file Table_3.DOCX]

| Table S3.  *Mediation Analyses for the association between Hierarchy and Alcohol Consumption in males and females.* | | | | |
| --- | --- | --- | --- | --- |
| Variable | R^2^ | *F* | β | *p* |
| 1. *Latitude* | .14 | 11.30 |  |  |
| Hierarchy |  |  | -.37 | .001 |
| 1. *Alcohol Male* | .12 | 9.99 |  |  |
| Latitude |  |  | .35 | .002 |
| 1. *Alcohol Male* | .12 | 10.15 |  |  |
| Hierarchy |  |  | -.35 | .002 |
| *c’. Alcohol Male* | .18 | 7.76 |  |  |
| Hierarchy |  |  | -.26 | .029 |
| Latitude |  |  | .25 | .031 |
| Sobel Test = -.09, *SE* = .05, *p* = .07 | | | | |
| 1. *Latitude* | .14 | 11.30 |  |  |
| Hierarchy |  |  | -.37 | .001 |
| 1. *Alcohol Female* | .14 | 11.24 |  |  |
| Latitude |  |  | .37 | .001 |
| 1. *Alcohol Female* | .31 | 32.27 |  |  |
| Hierarchy |  |  | -.56 | <.001 |
| *c’. Alcohol Female* | .34 | 18.29 |  |  |
| Hierarchy |  |  | -.49 | <.001 |
| Latitude |  |  | .19 | .07 |
| Sobel Test = -.07, *SE* = .04, *p* = .12 | | | | |
