# Supplementary material for: Cultural Value Orientations and Alcohol Consumption in 74 Countries: A Societal-Level Analysis
Source: Front Psychol. 2017 Nov 20;8:1963. doi: 10.3389/fpsyg.2017.01963 (PMC5702438; doi:10.3389/fpsyg.2017.01963)
Supplement: Supplementary file 4 [file Table_4.DOCX]

| Table S4.  *Mediation Analyses for the association between Mastery and Alcohol Consumption in males and females.* | | | | |
| --- | --- | --- | --- | --- |
| Variable | R^2^ | *F* | β | *p* |
| 1. *Latitude* | .01 | 0.63 |  |  |
| Mastery |  |  | -.09 | .429 |
| 1. *Alcohol Male* | .12 | 9.99 |  |  |
| Latitude |  |  | .35 | .002 |
| 1. *Alcohol Male* | .00 | .01 |  |  |
| Mastery |  |  | -.01 | .936 |
| *c’. Alcohol Male* | .12 | 4.94 |  |  |
| Mastery |  |  | .02 | .835 |
| Latitude |  |  | .35 | .002 |
| Sobel Test = -.09, *SE* = .05, *p* = .07 | | | | |
| 1. *Latitude* | .01 | 0.63 |  |  |
| Mastery |  |  | -.09 | .429 |
| 1. *Alcohol Female* | .14 | 11.24 |  |  |
| Latitude |  |  | .37 | .001 |
| 1. *Alcohol Female* | .01 | 0.76 |  |  |
| Mastery |  |  | -.10 | .387 |
| *c’. Alcohol Female* | .14 | 5.76 |  |  |
| Mastery |  |  | -.07 | .539 |
| Latitude |  |  | .36 | .002 |
| Sobel Test = -.03, *SE* = .05, p = .46 | | | | |
