# Supplementary material for: Cultural Value Orientations and Alcohol Consumption in 74 Countries: A Societal-Level Analysis
Source: Front Psychol. 2017 Nov 20;8:1963. doi: 10.3389/fpsyg.2017.01963 (PMC5702438; doi:10.3389/fpsyg.2017.01963)
Supplement: Supplementary file 5 [file Table_5.DOCX]

| Table S5.  *Mediation Analyses for the association between Affective Autonomy and Alcohol Consumption in males and females.* | | | | |
| --- | --- | --- | --- | --- |
| Variable | R^2^ | *F* | β | *p* |
| 1. *Latitude* | .13 | 11.20 |  |  |
| Aff. Autonomy |  |  | .37 | .001 |
| 1. *Alcohol Male* |  |  |  |  |
| Latitude |  |  |  |  |
| 1. *Alcohol Male* | .21 | 18.70 |  |  |
| Aff. Autonomy |  |  | .45 | <.001 |
| *c’. Alcohol Male* | .24 | 11.50 |  |  |
| Aff. Autonomy |  |  | .38 | .001 |
| Latitude |  |  | .21 | .061 |
| Sobel Test = .08, *SE* = .05, *p* = .11 | | | | |
| 1. *Latitude* | .13 | 11.20 |  |  |
| Aff. Autonomy |  |  | .37 | .001 |
| 1. *Alcohol Female* |  |  |  |  |
| Latitude |  |  |  |  |
| 1. *Alcohol Female* | .33 | 35.55 |  |  |
| Aff. Autonomy |  |  | .58 | <.001 |
| *c’. Alcohol Female* | .36 | 19.87 |  |  |
| Aff. Autonomy |  |  | .51 | <.001 |
| Latitude |  |  | .18 | .081 |
| Sobel Test = .07, *SE* = .04, p = .13 | | | | |
