# Supplementary material for: Cultural Value Orientations and Alcohol Consumption in 74 Countries: A Societal-Level Analysis
Source: Front Psychol. 2017 Nov 20;8:1963. doi: 10.3389/fpsyg.2017.01963 (PMC5702438; doi:10.3389/fpsyg.2017.01963)
Supplement: Supplementary file 6 [file Table_6.DOCX]

| Table S6.  *Mediation Analyses for the association between Intellectual Autonomy and Alcohol Consumption in males and females.* | | | | |
| --- | --- | --- | --- | --- |
| Variable | R^2^ | *F* | β | *p* |
| 1. *Latitude* | .24 | 22.51 |  |  |
| Intel. Autonomy |  |  | .49 | <.001 |
| 1. *Alcohol Male* |  |  |  |  |
| Latitude |  |  |  |  |
| 1. *Alcohol Male* | .16 | 13.82 |  |  |
| Intel. Autonomy |  |  | .40 | <.001 |
| *c’. Alcohol Male* | .19 | 8.43 |  |  |
| Intel. Autonomy |  |  | .30 | .016 |
| Latitude |  |  | .20 | .105 |
| Sobel Test = .10, *SE* = .06, *p* = .13 | | | | |
| 1. *Latitude* | .24 | 22.51 |  |  |
| Intel. Autonomy |  |  | .49 | <.001 |
| 1. *Alcohol Female* |  |  |  |  |
| Latitude |  |  |  |  |
| 1. *Alcohol Female* | .36 | 40.86 |  |  |
| Intel. Autonomy |  |  | .60 | <.001 |
| *c’. Alcohol Female* | .37 | 20.78 |  |  |
| Intel. Autonomy |  |  | .55 | <.001 |
| Latitude |  |  | .10 | .373 |
| Sobel Test = .05, *SE* = .05, *p* = .39 | | | | |
