# Supplementary material for: Cultural Value Orientations and Alcohol Consumption in 74 Countries: A Societal-Level Analysis
Source: Front Psychol. 2017 Nov 20;8:1963. doi: 10.3389/fpsyg.2017.01963 (PMC5702438; doi:10.3389/fpsyg.2017.01963)
Supplement: Supplementary file 7 [file Table_7.DOCX]

| Table S7.  *Mediation Analyses for the association between Egalitarianism and Alcohol Consumption in males and females.* | | | | |
| --- | --- | --- | --- | --- |
| Variable | R^2^ | *F* | β | *p* |
| 1. *Latitude* | .00 | 0.24 |  |  |
| Egalitarianism |  |  | .06 | .63 |
| 1. *Alcohol Male* |  |  |  |  |
| Latitude |  |  |  |  |
| 1. *Alcohol Male* | .00 | 0.33 |  |  |
| Egalitarianism |  |  | .07 | .565 |
| *c’. Alcohol Male* | .12 | 5.03 |  |  |
| Egalitarianism |  |  | .05 | .666 |
| Latitude |  |  | .35 | .003 |
| Sobel Test = .02, *SE* = .04, *p* = .65 | | | | |
| 1. *Latitude* | .00 | 0.24 |  |  |
| Egalitarianism |  |  | .06 | .63 |
| 1. *Alcohol Female* |  |  |  |  |
| Latitude |  |  |  |  |
| 1. *Alcohol Female* | .12 | 9.72 |  |  |
| Egalitarianism |  |  | .35 | .003 |
| *c’. Alcohol Female* | .24 | 11.23 |  |  |
| Egalitarianism |  |  | .33 | .003 |
| Latitude |  |  | .35 | .001 |
| Sobel Test = .01, *SE* = .04, *p* = .65 | | | | |
